# SWCNT Network-FET Device for Human Serum Albumin Detection

**DOI:** 10.3390/s22218212

**Published:** 2022-10-26

**Authors:** Iskandar Yahya, Muhamad Azuddin Hassan, Nur Nasyifa Mohd Maidin, Mohd Ambri Mohamed

**Affiliations:** 1 Faculty of Engineering & Built Environment, National University of Malaysia (UKM), Bangi 43600, Malaysia; 2 Institute of Microengineering and Nanoelectronics (IMEN), National University of Malaysia (UKM), Bangi 43600, Malaysia

**Keywords:** biosensor, carbon nanotubes, field-effect transistor, human serum albumin

## Abstract

A thin film of single-walled carbon nanotube (SWCNT) network field-effect transistor (FET) was fabricated by a simple, fast, and reliable deposition method for electronic applications. This study aims to develop a method for fabricating a thin film of random SWCNTs to be used as a transducer to detect human serum albumin (HSA) in biosensor applications. The random SWCNT network was deposited using the airbrush technique. The morphology of the CNT network was examined by utilising atomic force microscopy (AFM) and field-emission scanning electron microscopy (FESEM), while electrical characteristics were analysed using three-terminal IV measurements. The thin film (SWCNT network) was applied as a transducer to detect human serum albumin (HSA) based on its covalent interaction with antibodies. HSA plays a significant part in the physiological functions of the human body. The surface alteration of the SWCNTs was verified using Fourier transform infrared (FTIR) spectroscopy. Electrical current–voltage measurements validated the surface binding and HSA detection. The biosensor linearly recorded a 0.47 fg/mL limit of detection (LOD) and a high sensitivity of 3.44 μA (g/mL)^−1^ between 1 fg/mL and 10 pg/mL. This device can also be used to identify a genuine HSA despite interference from other biomolecules (i.e., bovine serum albumin (BSA)), thus demonstrating the random SWCNT-FET immunosensor ability to quantify HSA in a complex biological environment.

## 1. Introduction

Single-walled carbon nanotubes (SWCNTs) exhibit remarkable physical properties, such as optimum mobility of 100,000 cm^2^/Vs with 109 A/cm^2^ current carrying capacities, making them valuable in various electronic applications. In addition, they also have up to a 10^6^ *I_on_/I_off_* current ratio. Although preferable in some ways, single nanotube applications suffer primarily from irreproducibility caused by the geometry differences between tubes, in addition to chirality disparities, which has presented challenges in repeated reproduction of predictable devices. Hence, a low-cost and highly efficient method for continuous mass production of thin film (random SWCNT network) for many different applications is appropriate [1,2].

Direct exposure of thin film carbon materials in SWCNT FET-based biosensors to the environment [3] can cause slight interface changes, leading to considerable electrical characteristic modifications of nanomaterials [4,5], which increases the sensitivity of biosensing. Intermolecular interactions among biomolecules between the carbon nanomaterial interface and the environment significantly modify the SWCNT nanomaterial electrical characteristics in FET biosensors through the following mechanisms: (1) electrostatic gating or surface charge-induced gating, (2) transfer of charges between biomolecules and carbon nanomaterials, and (3) scattered charges throughout the carbon nanomaterial interface [6].

Optical detection and immunoassay are widely used in biological sensing systems. Although these techniques are highly sensitive and selective, they are also fundamentally complex due to the numerous steps involved between the engagement of the analyte and the signal creation [7]. Additionally, these approaches also require multiple reagents, preparative processes, signal amplification, and complicated data processing. While single-molecule detection has been performed in a few circumstances, the method necessitates the use of optical probe molecules which may alter the biomolecule activity [8]. Meanwhile, electronic detection, which makes use of nanoscale devices, has two key advantages. The first advantage of this electronic detection technique is compatibility in terms of size. The second benefit is that most biological processes, including electrostatic interactions and charge transfer, allow electronic detection and eventually the integration of biology with electronics. Due to these two advantages, SWCNT thin film is applicable as a transducer for biosensor applications.

Human serum albumin (HSA) represents the largest protein fraction in human blood plasma as required for life. It contains about half of the protein in blood serum [9], having a dimension of 7.5 × 6.5 × 4.0 nm^3^ with a molecular weight of Mw = 66.4 kDa [10]. HSA performs several crucial physiological activities, including regulating oncotic pressure, balancing blood pH, as well as delivering hormones, fatty acids, medications, and other vital compounds through the bloodstream [11]. The HSA concentration is regularly evaluated in the basic metabolic panel which represents a person’s general health state [12]. For diabetes, which is a metabolic and endocrine illness that harms human health, albumin levels in the urine are a key sign for early detection of diabetic nephropathy, which is a chronic diabetic consequence [13]. Albuminuria, or high protein levels in the urine (20–200 mgL^−1^), can indicate renal failure [14]. Hence, it is critically important to develop a rapid and accurate detection method in the quantitative analysis of HSA detection. Due to the lack of approaches in terms of sensitivity and specificity in existing studies, the current study identified the need to design a novel SWCNT-FET immunosensor to detect HSA (at concentrations between 1 fg/mL and 10 pg/mL).

Some recent work has been completed with SWCNT-FET biosensors to detect albumin [15], DNA [16,17], alpha-fetoprotein [18], glutamate [19], and COVID-19 [20]. However, this study aims to present the fabrication of an SWCNT-FET device using the airbrush technique to obtain a monolayer thin film which enhances sensitivity and specificity in detecting HSA. This deposition method was essential not only for HSA detection but also for other applications in clinical care and biochemical investigations.

## 2. Materials and Methods

### 2.1. Preparation of Substrates

Two types of substrates, namely glass and Si wafer, were used with thermally grown SiO_2_. Substrates with a back-gate and a silicon wafer (p-type, 1–10 ohm.com) applied with a wet-thermally generated SiO_2_ layer (300-nm thick) were used, in which the SiO_2_ layer served as the gate oxide. The wafer was cut into individual dies with a size of approximately 2 cm × 2 cm. Removal of organic and inorganic contaminants in the substrates was performed with an ultrasonic bath using acetone, isopropyl alcohol (IPA), and deionised water (DIW) for 10 min, respectively.

### 2.2. SWCNT Thin Film Deposition

Two types of SWCNT solutions were prepared in this study. In the first solution, 1 mg of direct injection pyrolytic synthesis (DIPS) SWCNTs (70% semiconducting, 0.8–3 nm diameter, Nikkiso Japan) was mixed with 20 mL of deionised water (DIW) and 1% TX-100 (Sigma Aldrich, St. Louis, MO, USA) to form a stable solution. The TX-100 acts as a surfactant to promote de-bundling and dispersion. Then, the solution was physically agitated using tip-probe ultrasonication (300 V/T, ultrasonic homogeniser, Biologist Inc., Manassas, VA, USA) in an ice bath for two hours at 90% power using a tip 300 V/T ultrasonic homogeniser. The second solution was repeated by replacing DIPS SWCNTs with cobalt and molybdenum oxide catalyst (CoMoCAT) SG 65 SWCNTs (90% semiconducting, 0.7–0.9 nm diameter, Sigma Aldrich, USA). Both samples were grown based on chemical vapour deposition (CVD) process. Although DIPS SWCNTs were known to be less suitable than CoMoCAT SWCNTs for building a FET device because of their higher metallic content, they were also considered in this study in order to establish the conditions for the SWCNT deposition process due to lower cost and availability. The CoMoCAT SG 65 SWCNTs samples were sourced from an optimised growth process that selectively, up to 90% purity, produces (6,5) SWCNTs, which are semiconducting. It was therefore more economical to optimise the spray coating parameters using much lower-cost DIPS SWCNTs that have similar physical properties before dispersion in DIW.

SWCNTs were then deposited using the airbrush technique with optimised nozzle distance, air pressure, substrate temperature, and duration. Then, the substrates were placed on a hotplate for soft baking for five minutes at 100 °C before light washing with DIW and hard baked at 200 °C for 15 min. The process for the CoMoCAT SG 65 SWCNTs needs to ensure that a complete linkage network is achieved, otherwise, the deposition (spray) process needs to be repeated. This process is quite critical to control the SWCNT thin layer in order to avoid metallic behaviour that can adversely affect device performance.

The thin film surface morphology was examined by characterising the SWCNT thin film using the non-contact mode AFM (NX-10 by Park System, Suwon, Korea) with a 10 nm cantilever tip SPA400 and FESEM (Zeiss Supra 55VP, Jena, Germany).

### 2.3. Fabrication of the SWCNT Network Device

**Figure 1:**
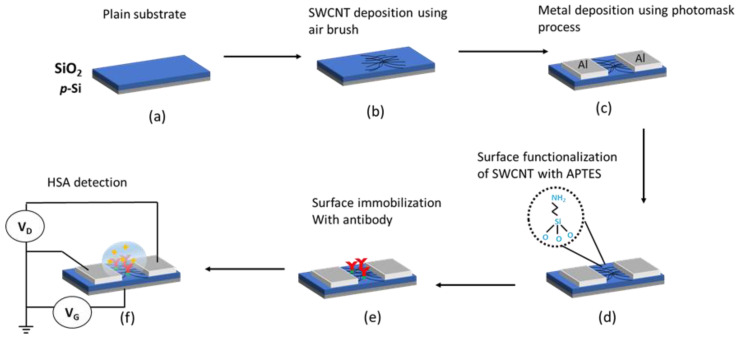
A schematic fabrication process of the CNT-FET device; (**a**) cleaned substrate, (**b**) SWCNTs deposition, (**c**) Al metal deposition, (**d**) surface functionalisation, (**e**) surface immobilisation with Ab, and (**f**) electrical measurement.

A conventional commercial airbrush nozzle at a pressure of 8.0 psi [21], covered by a shadow mask (0.5 mm channel), was used to deposit the SWCNT solutions on the substrates above a hotplate at 80 °C (Figure 1). The hotplate was required to speed up the solution evaporation, allowing the SWCNT network to be attached to the desired surface area without bundling due to movements from the fluid surface tension. Next, the substrates were soft baked at 100 °C for five minutes to improve cohesiveness, and then washed using DIW to remove surfactant, dried with compressed N_2_, and hard baked at 200 °C for 15 min. Finally, aluminium (Al) electrodes were deposited on the substrates using thermal evaporation (Torr, Auburn, WA, USA) directly on the SWCNT thin film forming top contacts (electrodes contacting the top of SWCNTs) as shown in Figure 1b,c. The Al electrodes form direct ohmic contacts with the SWCNTs in an ultra-high vacuum chamber with virtually no spontaneous formation of Al_2_O_3_ in between them at the contact surface. Upon removal from the chamber, the top surface of the Al electrodes contact pads however may consist of Al_2_O_3_ layer that needs to be penetrated by the measurement probes during the electrical characterisation step to minimise contact resistance.

The measurement of 2-terminal probes (Keithley 2635B) was performed to ensure the development of SWCNTs percolation between both electrodes. The deposition process requires repetition if no current is available.

### 2.4. SWCNTs Surface Functionalisation, Detection and Characterisation

The transducer was functionalised by incubating it with 0.3 µL of 2% 3-aminopropyltriethoxysilane (APTES) (99%, Sigma-Aldrich, St. Louis, MO, USA). The unbound APTES was later removed by rinsing the transducer with DIW thrice, followed by immobilising 0.3 μL of albumin antibody (antihuman albumin antibody generated in rabbit, Ab, Sigma-Aldrich) onto the APTES-functionalised SWCNTs surface, before being incubated at ambient temperature for 30 min. The elimination of any unbound antibodies was completed by rinsing the antibody-modified CNTs with DIW.

The HSA samples were sourced from Sigma-Aldrich in the form of 50 mg/mL H_2_O soluble solution (HSA, >99 percent). The HSA solution was diluted by adding DIW at a specified ratio of 100 μL HSA + 900 μL DIW to obtain 1 mg/mL solution, and then DIW kept being added to obtain lower concentrations. For this experiment, HSA was detected by incubating 0.3 μL of different HSA concentrations (1 fg/mL, 10 fg/mL, 100 fg/mL, 1 pg/mL, and 10 pg/mL) (HSA, >99 percent, Sigma-Aldrich) at ambient temperature for 30 min [22] to enable HSA-antibody (Ab) bonds. For the selectivity test, 0.3 μL of 1 fg/mL and 10 pg/mL bovine serum albumin (BSA > 96 per cent, Sigma-Aldrich, USA) were incubated for 30 min, respectively. The covalent binding interactions between antibody and the HSA target, which caused a change in the drain current (*I_D_*) of the constructed biosensor, are the basis for the detection mechanism. The Fourier transform infrared (FTIR) spectroscopy (Spectrum 400 FT-IR; PerkinElmer, Waltham, MA, USA) was used to analyse the functional groups on the device transducer surface after the APTES and Ab functionalisation.

### 2.5. FET-Based Biosensor Electrical Characterisation

The biosensor electrical characterisation was conducted using a three-probe system, the Keithley 2635B. A sweep gate voltage (*V_G_*) between −5 and 5 V supplied the drain electrode of the device by grounding the source electrode and supplying a *V_D_* of 5 V bias to the substrate–source electrode. The electrical measurement of the thin film conductivity was performed using a semiconductor parametric analyser (SPA) (Keithley 2635B; Keithley Instruments, Cleveland, OH, USA). The *I_D_-V_G_* graph of the initial device was plotted based on three setup parameters: (1) without surface functionalisation; (2) after surface immobilisation with Ab-HSA; and (3) with various HSA biomarker concentrations (1 fg/mL, 10 fg/mL, 100 fg/mL, 1 pg/mL, and 10 pg/mL).

Every electrical measurement to detect HSA can only be performed after a complete incubation and washing of the device to avoid non-specific binding [23]. Therefore, the characterisation process only involved the Ab-HSA biomarker immobilised on the transducer surface. A device channel length of 0.5 mm was chosen for FET measurement, which is addressed in the next section. Figure 1 shows the schematic fabrication process of the SWCNT-FET network construction for quantifying HSA.

## 3. Results

### 3.1. Thin Film Characterisation

**Figure 2:**
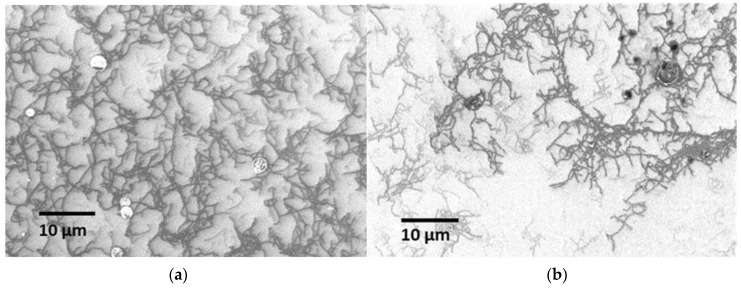
Surface morphology of DIPS SWCNT thin film using FESEM at 1000Χ with substrate–nozzle distance at: (**a**) 10 mm; (**b**) 4 mm.

The DIPS SWCNTs (70% semiconducting) and CNT-FET CoMoCAT SWCNTs (90% semiconducting) were chosen in the study of the SWCNT thin film [24]. The primary target of depositing the SWCNTs was to develop a thin film. An artist’s airbrush was used to ensure only a diminutive quantity of water (mist) was dropped on the substrates to minimise the “coffee ring” effect [25]. The substrates were also heated at 80 °C to ensure quick evaporation of the small drop of water once it hit the surface [26]. A shadow mask with a 0.5 mm opening was used to secure the device focus point for a smooth operation without photolithography processes or a clean room. Optimising the thin film, in particular by obtaining a good dispersion of the SWCNTs, was completed by adjusting the substrate–nozzle distance [27]. In this work, the substrate–nozzle distance was optimised at 10 mm, as shown in the FESEM analysis of the DIPS SWCNTs sample (Figure 2). Electrical measurements also confirmed this optimisation result, in which the 10 mm distance produced the highest current at 1V (15.30 µA), followed by the distance of 13 mm (10.30 µA), 7 mm (8.82 µA), and 4 mm (3.2 µA). In this experiment, three or more sprays were performed to obtain a good SWCNT network.

Although the low-density SWCNT network consists of individual routes rather than bulk material, the electrical behaviour of a single device is determined not only by the shape of the thin film but also by the network density [28,29]. As a consequence, equivalent electrical characteristics are achievable for various shapes. All this indicates that the density of the SWCNT network must be carefully managed to obtain repeatable results [30].

The standard percolation theory describes the density dependence of conductivity as:

σ ∝ (*N* − *N_c_*)*^α^* (1)

**Figure 3:**
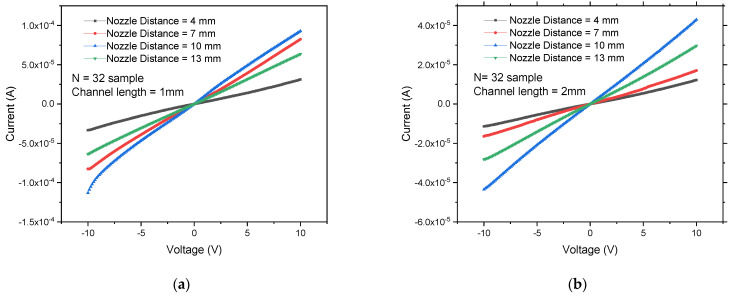
IV measurements for studying substrate–nozzle distances using DIPS SWCNTs sample at different channel length; (**a**) 1 mm; (**b**) 2 mm.

*N* is the conducting stick (CS) density, while *N_c_* refers to the corresponding critical density with percolation threshold. By referring to the three-dimensional conductivity and two-dimensional sheet conductance (G), N signifies the CS density while N_c_ represents the critical density affiliated with the percolation threshold [1]. The CNT aspect ratio significantly impacts the entanglement criterion: as the aspect ratio drops, the conductivity loss induced by the entanglement effect becomes more critical [31]. Generally, increasing CNT agglomeration leads to reduced current output, as shown in Figure 3. The highest current was consistently recorded by the least agglomeration occurring at 10 mm substrate–nozzle distance, followed by 7 mm, 13 mm, and finally, 4 mm which shows the highest agglomeration.

This result suggests that the optimisation nozzle distance at 10 mm is the best output for the CNT-FET device. A high on-current (*I_on_*) is resulted from a higher-density network, whereas a high off-current (*I_off_*) results from more metallic CNTs, thus producing a low on/off ratio. In this study’s experiment, more than five sprays caused the *I_on_/I_off_* ratio to drop due to the involvement of metallic SWCNTs. In an early study, the SWCNTs concentration in solution was studied to obtain the best random network FET performance [32].

**Figure 4:**
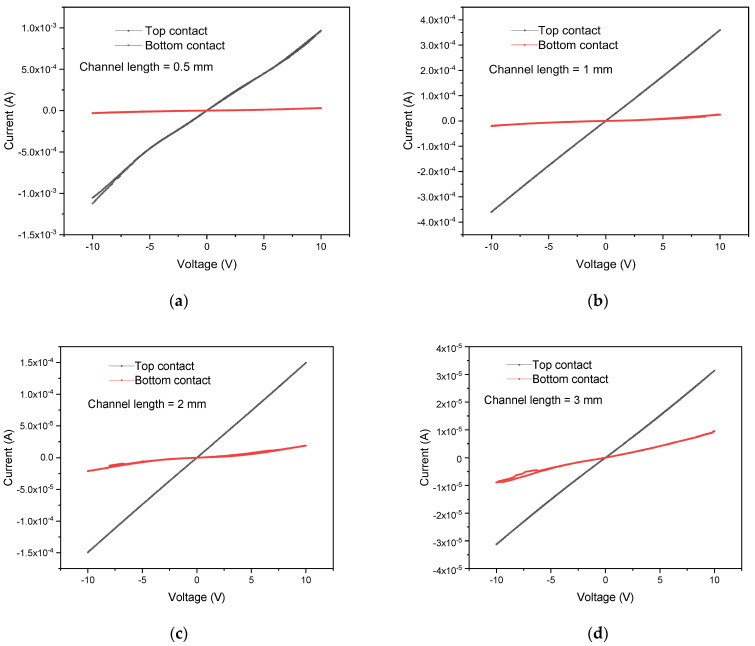
IV results for bottom and top contacts with DIPS SWCNTs at different channel lengths; (**a**) 0.5 mm; (**b**) 1 mm; (**c**) 2 mm; (**d**) 3 mm.

In addition to the network density, the SWCNT contact between electrodes is also essential to develop a good device for HSA detection due to floating CNTs, contact resistance, and others. Based on the experimental findings, choosing to have the SWCNT thin film in top contact configuration, i.e., depositing SWCNTs before the electrodes were justified based on the recorded high conductance as shown in Figure 4. Furthermore, unlike top contact, bottom contact requires special treatment to improve the conductivity [33], such as pre-transfer electrodes and post-transfer annealing. Another factor that affects the performance of bottom contact is contact resistance [34] between electrodes and SWCNTs due to the development of an aluminium oxide layer [35] once it is exposed to ambient air. Figure 4 shows the consistently high current during the experiment of DIPS SWCNTs at 0.5, 1, 2, and 3 mm channel lengths.

**Figure 5:**
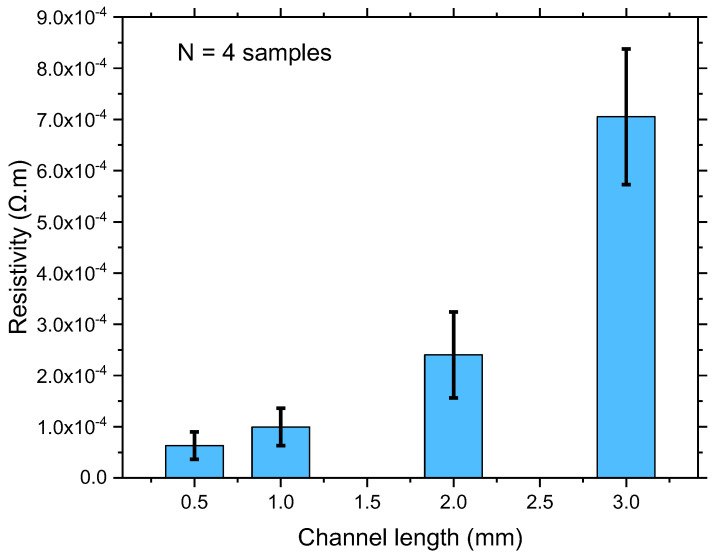
Comparison of the resistivity standard deviations at 0.5, 1, 2, and 3 mm channel lengths using CoMoCAT SG 65 SWCNTs.

To ensure a good dispersion of the SWCNT network deposition, the resistivity standard deviation at four different channel lengths was analysed, and results are illustrated in Figure 5. As observed, the resistivity of the CNT thin film consistently increased from 0.5 mm to 3 mm channel lengths, which is relevant according to Equation (2). This indicates that the shortest channel length is the most suitable setting for this device.

Resistivity, *ρ* = *RA*/*L*; *A* = *W* × *h*; (2)

A CoMoCAT SG 65 SWCNT thin film has a chirality (6,5) semiconducting type (90% semiconducting, Sigma-Aldrich, USA). In this experiment, the SWCNT network has to be a semiconducting enriched type to produce a FET. Low density prevents the formation of continuous path throughout the surface; hence, the conductance (*G*) is zero. The thin film achieves a critical density (*N_c_*, also known as the percolation threshold) as the density increases, at which routes start to form. According to the theory, the conductance of a percolating network should vary at a critical density, as given in Equation (3):

*G* ∼ (*N* − *N_c_*)*^α^* (3)

The critical density, *N_c_*, is determined by the geometry of the networked object based on the theoretical simulations for an identical length stick network (*L_T_*), as given in Equation (4) [24]:

*N_c_* = 5.7/*L_T_*^2^ (4)

**Figure 6:**
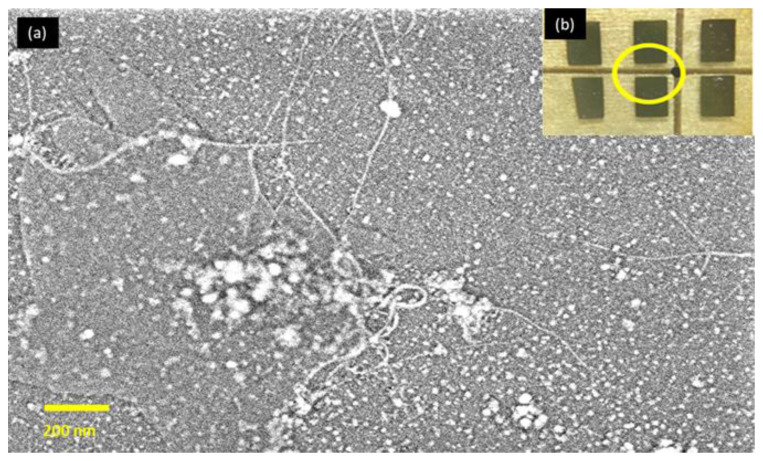
The surface morphology of randomly selected spot for a CoMoCAT SG 65 SWCNTs sample: (**a**) FESEM image with 50k magnification; (**b**) the yellow circle is the scan location on the substrates that contain SWCNT network. Note that the presented nanoparticles on the surface were most likely from the deposited 2 nm thick Pt layer to avoid electrostatic charging during FESEM measurements.

Employing higher aspect ratio materials is beneficial to create percolating routes at a considerably lower density, nearing *N_c_* = 0, for genuinely one-dimensional (1-D) objects. The critical element (*α*) relies entirely on the percolating space dimension, unlike the critical density *N_c_* [1]. This revelation implicates composite creation, including transparent applications of the nanotubes [32]. Figure 6a shows the morphology scan of the SWCNTs sample by FESEM (Zeiss Supra 55VP, Germany), and Figure 6b shows the location of the scanned thin film. These results show the dispersion of CoMoCAT SG 65 SWCNTs at the monolayer to allow percolation in great conditions.

**Figure 7:**
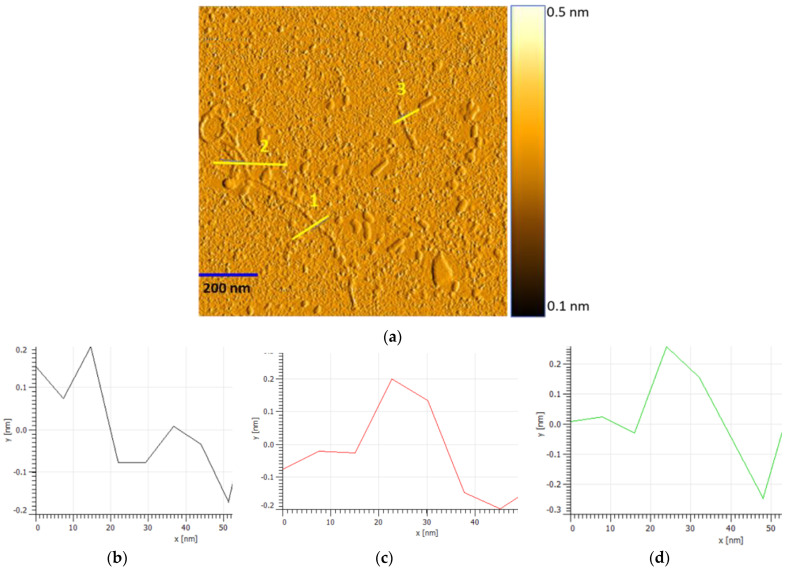
(**a**): AFM image of the CoMoCAT SG 65 SWCNTs with a diameter between 0.7 nm–0.9 nm. 1, 2 and 3 are the measured line profiles; (**b**–**d**): measured AFM line profiles with average tube diameter ~0.5 nm for profiles 1, 2 and 3, respectively.

AFM is a crucial tool for performing nanometre-scale imaging. It was used to obtain an accurate profile or diameter (*d_t_*) of the tube height for randomly selected individual SWCNT and SWCNT bundles [36]. Figure 7a shows the surface morphology of the CoMoCAT SG 65 SWCNTs, and Figure 7b–d presents the measured AFM line profiles showing a tube diameter of around 0.5 nm. Compared to DIP SWCNTs, the CoMoCAT SG 65 SWCNTs have a slightly different diameter specification labelled between 0.7 nm–0.9 nm.

### 3.2. Electrical Characteristics of Network SWCNT-FET

The biosensor device utilises the CoMoCAT SG 65 SWCNT film due to its 90% semiconducting materials. This is unlike the DIPS SWCNT film which has only 70% semiconducting materials and 30% metallic. The possibility of producing a high *I_on_/I_off_* ratio is difficult if metallic nanotubes are present in the link network. Distinct length scales in a random CNT-FET such as screening length, channel length, and mean free path, would influence the comprehensive behaviour of drive voltage responses, much as they do for CMOS transistors [37].

The device channel length is 500 µm (0.5 mm), which is quite long compared to ordinary transistors with about 0.5–100 µm length. However, the key benefit of the use of a random SWCNT network as presented in this study is the transistor’s improved performance in terms of on-current, on/off ratio, and transconductance [38]. Furthermore, a large channel length would produce a good device since it is easier, requires a lower cost, and produces faster than the smaller channel device [39].

**Figure 8:**
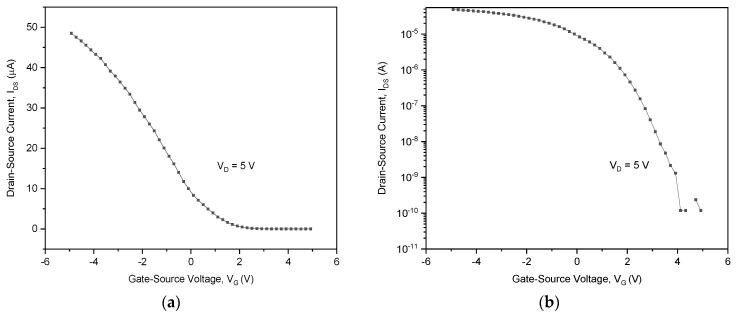
Electrical characterisation results of the CoMoCAT SG 65 SWCNTs: (**a**) transfer characteristics; (**b**) *I_on_/I_off_* ratio is ~10^5^.

The transfer characteristics (*I_D_* vs. *V_G_* at constant *V_D_*) and output characteristics (*I_D_* vs. *V_D_* at constant *V_G_*) exhibited the *I_on_/I_off_* ratio which is greater than 10^5^, as shown by the transfer graph in Figure 8. In detail, the transconductance is 9.85 μS, the threshold voltage is 2.5 V [40], the subthreshold voltage is 4.6 × 10^6^ with 0.6 V/decade, and mobility = 176 Cm^2^/Vs. Overall, the transfer graph shows a very effective SWCNT-FET network.

The following section is divided into sub-headings to concisely and precisely describe the experimental results, their interpretations, and concluding remarks.

### 3.3. Characterisation of Surface Functionalisation

**Figure 9:**
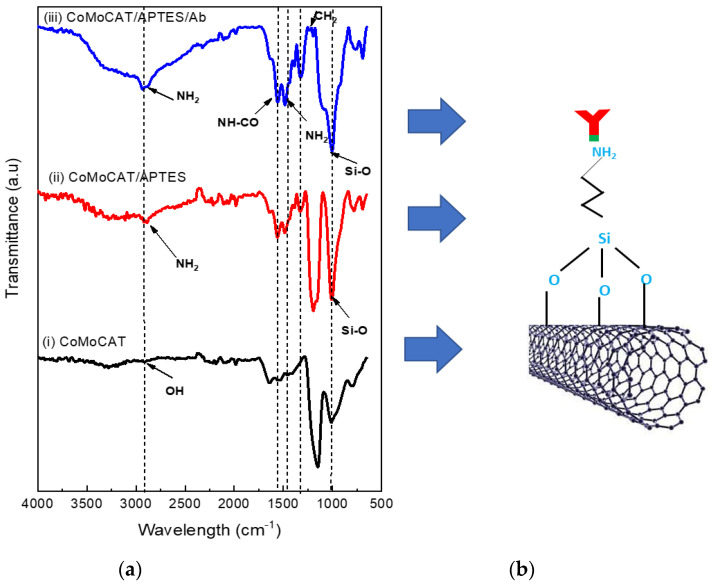
Surface functionalisation characteristics of the CoMoCAT SG 65 SWCNT thin-film network; (**a**) FTIR spectra absorption of (**i**) bare SWCNTs, (**ii**) SWCNTs functionalised with APTES, (**iii**) APTES-functionalised-SWCNTs immobilised with Ab; (**b**) an illustration of the Ab/APTES-modified SWCNTs.

Figure 9 presents the emerging functional groups on the SWCNTs surface post-modification with APTES and antibodies. The hydroxyl (–OH) group produced from the raw surface [41], as seen in Figure 9a(i), has a mild stretching vibration band at 2900 cm^−1^. After being functionalised with APTES, the absorption intensity of the 2900 cm^−1^ peak corresponding to the amine (–NH) group increased (Figure 9a(ii)), while a less-intense shoulder at the 1000 cm^−1^ peak signified the Si-O group yield.

These results indicated that the amino silane linkage was successfully formed on the surface, which was also in perfect consensus with an earlier study [42]. The Ab immobilisation on the APTES increased the amine group, which was indicated by an increased absorption intensity at 2900 cm^−1^ (Figure 9a(iii)), attributed to the covalent interaction between the amine group on both the APTES and Ab.

The C=O stretching in the amide’s linkage created the amide I band which vibrated at 1650 cm^−1^, while the bending vibrations of Ab’s amine end functional group produced the amide II band that vibrated at 1545 cm^−1^. The 1320 cm^−1^ peak could be due to the C–H bond bending vibrations from Ab. In addition, the resulting 1130 cm^−1^ peak can also be attributed to the Si–O bond stretching vibration. All of the attributed peaks are in line with the results in a previous study [43], indicating the Ab immobilisation on the SWCNTs surface.

### 3.4. Detection of HSA

**Figure 10:**
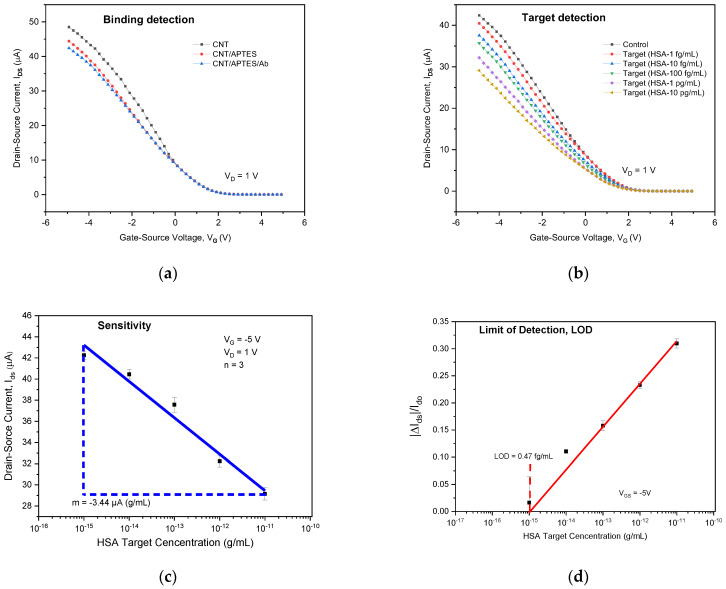
Electrical detection characteristics of the random SWCNT-FET (CoMoCAT type) biosensor for HSA: (**a**) transfer curve of the SWCNT-FET for binding detection with APTES and Ab; (**b**) transfer curve of the SWCNT-FET in response to the HSA target (1 fg/mL to 10 pg/mL); (**c**) *I_D_* response curve of the biosensor at different HSA target concentrations whereby the blue line shows the curve fitting for a linear drain current in the varying HSA target concentration at 3.44 μA.(g/mL)^−1^ (380/decade of change in the HSA target); (**d**) the calibration curve of the relative change in *I_D_* determines the LOD where *I_do_* = 4.26 × 10^−5^ μA, in which *I_do_* is the base current in the applied antibody before varying HSA concentrations were introduced.

Each antibody–antigen binding in the CNT-FET immunological sensing system shifts the current in the transfer characteristic. As illustrated in Figure 10a, the APTES-functionalised SWCNTs had a lower *I_D_* than the raw SWCNTs. The I_D_ was slightly reduced during the Ab immobilisation on the APTES-functionalised SWCNTs. With the addition of 1 fg/mL and 10 pg/mL concentrations, the transfer curves provide more information on the CNT-performance FETs in measuring the HSA target. Figure 10b shows the *I_D_* proportional increases to the HSA target concentrations. The improved antibody immobilisation caused more protein binding interactions, resulting in a higher measured *I_D_* of the biosensor.

During binding contacts, it is more plausible for a negatively charged HSA to transmit the charge to the transducer [15]. This also suggests that a high SWCNT aspect ratio offers a greater surface area during antibody immobilisation, and additionally, it facilitates the transporting of electrons as a transducer. Figure 10c presents the *I_D_* response curve of the biosensor at various HSA target concentrations. HSA concentrations are satisfactorily directly proportional between 1 fg/mL and 10 pg/mL at 3.44 μA·(g/mL)^−1^ sensitivity (380/decade of change in the HSA target). The limit of detection (LOD) was also investigated, as shown in Figure 10d.

Based on the standard deviation of the responses and the slope of the calibration curve, the relative change in the drain current (*I_D_*) in the biosensor exhibits a linear relationship across the HSA logarithmic concentrations between 1 fg/mL and 10 pg/mL at 0.47 fg/mL of the LOD. By comparing to the literature studies, the constructed immunosensor LOD and sensitivity range results obtained in this study were both considered excellent.

Table 1 compares the analytical performance of the SWCNT-FET device with other materials and measuring methods used in previous studies. In one study, the HSA detection utilising electrochemical polymers and polymer sensors with molecular imprint substrate was assessed using electrochemical impedance spectroscopy (EIS) with a detection limit of 16.5 ng/mL and 800 ng/mL, respectively [9]. In another study [7], Au nanoparticles were placed on a substrate with an antibody as the sensing probe could detect HSA concentrations at the lowest value of 0.01 μg/mL. Meanwhile, Kim et al. [15] who studied an SWCNT network with π-π interaction used the CNT-FET device and measured a LOD of 18.6 ng/mL. Another study [9] which demonstrated the HSA sensing using the chemiluminescence method with an Ultramicro CL probe achieved a LOD as low as 17.32 ng/mL. Siti et al. [44] who used ZnO NRs on a Zn NRs-FET device reported a LOD of 9.81 ng/mL with covalent bonding. As evidenced, the LOD of the HSA biosensor produced in this study is lower than that of other field-effect transistor-based biosensors as reported by previous studies.

**Figure 11:**
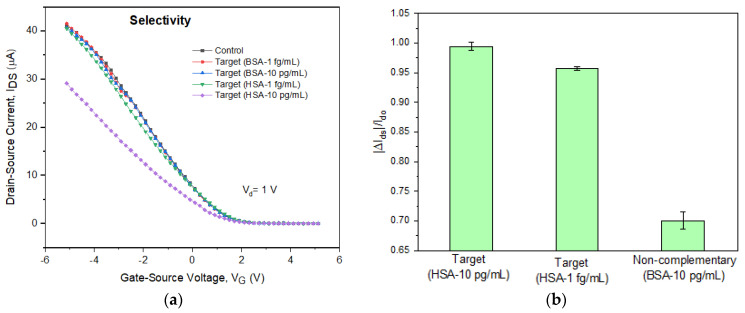
Selectivity detection: (**a**) toward HSA targets (1 fg/mL and 10 pg/mL) and non-complementary BSA targets (1 fg/mL and 10 pg/mL); (**b**) bar graph of the gate-source voltage response to specific targets and non-specific binding (using CoMoCAT SWCNTs).

As illustrated in Figure 11, the biosensor developed in this study exhibits a considerable I_D_ change of 0.99 ± 0.03 and 0.96 ± 0.01, respectively, upon the 10 pg/mL and 1 fg/mL of HSA targets. On the other hand, the non-complementary BSA target of 10 pg/mL displays practically no change in *I_D_*. This result indicates that the device detected HSA rather than BSA by performing a specific binding.

## 4. Conclusions

Random network-based SWCNT-FETs were fabricated in this study by using a simple, fast, and reliable solution-based process. A thin film of random SWCNTs can be developed as a good and powerful transducer applicable in biosensing devices and applications, including for HSA detection. The current study has demonstrated several capabilities of SWCNTs, including electrical conductivity, surface morphology and functionalisation, as well as gating effect, in addition to the final FET integration for HSA target detection. Additionally, the biosensor *I_D_* was reduced efficiently through a specific HSA target-antibody binding interaction. The biosensor responsiveness was tested by measuring the change in *I_D_* as HSA concentrations increased, which led to a sensitivity of 3.44 μA.(g/mL)^−1^ with 0.47 fg/mL LOD. The SWCNTs-enhanced surface area was found to be able to improve the covalent binding contacts in bio-recognition activities, enabling the biosensor to identify HSA in a complicated biological structure. This study provides useful findings for the future development of FET biosensors for other biomarkers and applications in clinical care and biochemical investigations.

## Figures and Tables

**Table 1:** Performance of HSA detection in this study in comparison to previous immunosensor studies.

Sensing Technique	Linear Range	Detection Limit	Reference
Chemosensor based on semi-covalent imprinting	0.8 μg/mL–20 μg/mL	16.5 ng/mL	[9]
Chemosensor based on semi-covalent imprinting	4 μg/mL–800 μg/mL	800 ng/mL	[9]
Wireless magnetoelastic (ME) immunosensor based on Au nanoparticle	0.01 μg/mL−100 μg/mL	0.01 μg/mL	[7]
CNT-FET	0.07 μg/mL–70 μg/mL	18.6 ng/mL	[15]
Chemiluminescence intensity	0.05 μM/L–1.5 μM/L	17.32 ng/mL	[45]
ZnO NRs-FET	0.01 μg/mL–100 μg/mL	9.81 ng/mL	[44]
CNT-FET based on the SWCNT network	1 fg/mL–10 pg/mL	0.47 fg/mL	This work
